# Spatio-temporal expression patterns of glycine-rich beta proteins and cysteine-rich beta proteins in setae development of *Gekko japonicus*

**DOI:** 10.1186/s12864-024-10426-8

**Published:** 2024-05-31

**Authors:** Longjie Xia, Chao Li, Shengnan Chen, Linna Lyu, Wenli Xie, Jie Yan, Kaiya Zhou, Peng Li

**Affiliations:** Herpetological Research Center, College of Life Sciences, Nanjing Normal University, Nanjing, Jiangsu 210023 P. R. China

**Keywords:** *Gekko japonicus*, Embryo development, Cysteine-rich corneous beta proteins, Glycine-rich corneous beta proteins, RNA-seq

## Abstract

**Background:**

Setae on the pad lamellae of the Japanese gecko *Gekko japonicus* (Schlegel, 1836), a vital epidermal derivative, are primarily composed of cornified beta-proteins (CBPs) and play a pivotal role in adhesion and climbing. The amino acid composition of CBPs might be a determining factor influencing their functional properties. However, the molecular mechanisms governed by *CBP* genes with diverse amino acid compositions in setae development remain unexplored.

**Results:**

Based on RNA-seq analyses, this study confirmed that all *G. japonicus* CBPs (GjCBPs) are involved in setae formation. Cysteine-rich CBPs encoding genes (*ge-cprp-17* to *ge-cprp-26*) and glycine-rich CBPs encoding genes (*ge-gprp-17* to *ge-gprp-22*) were haphazardly selected, with quantitative real-time PCR revealing their expression patterns in embryonic pad lamellae and dorsal epidermis. It is inferred that glycine-rich CBPs are integral to the formation of both dorsal scales and lamellar setae, cysteine-rich CBPs are primarily associated with setae development. Additionally, fluorescence in situ hybridization revealed spatiotemporal differences in the expression of a glycine-rich CBP encoding gene (*ge-gprp-19*) and a cysteine-rich CBP encoding gene (*ge-cprp-17*) during dorsal scales and/or lamellar development.

**Conclusions:**

All 66 CBPs are involved in the formation of setae. Glycine-rich CBPs hold a significant role in the development of dorsal scales and lamellar setae, whereas most cysteine-rich CBPs appear to be essential components of *G. japonicus* setae. Even GjCBPs with similar amino acid compositions may play diverse functions. The clear spatio-temporal expression differences between the glycine-rich and cysteine-rich CBP encoding genes during epidermal scale and/or setae formation were observed. Embryonic developmental stages 39 to 42 emerged as crucial phases for setae development. These findings lay the groundwork for deeper investigation into the function of GjCBPs in the development of *G. japonicus* setae.

**Supplementary Information:**

The online version contains supplementary material available at 10.1186/s12864-024-10426-8.

## Background

As reptiles and birds evolved from ancestors, they adapted to terrestrial environments primarily by developing cornified skin, which offers protection against mechanical damage, ultraviolet radiation, and water loss, and through amniotic egg reproduction [1]. In the course of their adaptation to terrestrial life, they have developed a variety of epidermal derivatives such as bird feathers, reptile scales, claws and setae [2–4]. These unique features have facilitated the adaptation of reptiles and birds to a myriad of ecological niches that align with their distinct lifestyles [5–8]. The functions of these highly specialized epidermal derivatives vary considerably [9, 10]. For instance, feathers serve purposes of flight and insulation, scales offer body protection, and claws and setae aid in climbing. Setae, in particular, have garnered significant attention due to their unique function [11]. Positioned on the pad lamellae of certain reptiles [12–14], these setae empower them to navigate vertical and inverted surfaces [15–18]. They showcase a spatula-like structure, arising from the cytoskeletal organization and mechanical separation of keratin bundles [19–21]. While it is crucial to study the morphological changes of setae during embryonic development, the research on the development of setae or entire lamellae are very scarce and limited to few species [22–26]. Their functionality is derived from a combination of mechanisms operating either independently or collectively under specific environmental and substrate contexts [5, 27–31]. While the morphological characteristics of setae and spatulae account for some of their adhesive properties, understanding their chemical composition and conformation remains crucial.

Setae, along with other mechanically resistant skin barriers and appendages of reptiles, including snake scales, lizard setae and claws, and turtle carapaces, are derived from the products of corneous beta-proteins (CBPs) [11, 32–34]. Exclusive to reptiles and birds [35–38], CBPs, previously termed as beta-keratins, serve as a distinctive feature setting birds and reptiles apart from other vertebrates [9, 39]. The inherent structure of CBPs facilitates reptiles and birds to adapt to a plethora of ecological niches based on their specific lifestyles [4, 6–8]. During cornification and/or keratinization, CBPs progressively amass in the upper differentiating layers of the epidermis in birds and reptiles. This accumulation leads to the formation of a rigid corneous beta-material, culminating in a dense, compact corneous beta-layer [10, 40]. In -depth studies have already been conducted on CBPs in the setae of numerous reptiles [9, 41], delving into the role of CBPs in setae could enhance our comprehension of adhesive mechanisms.

The amino acid composition of CBPs is postulated to significantly affect the characteristics of epidermal derivatives. In a lizard *Anolis carolinensis*, 40 CBPs were identified and subsequently categorized into four distinct subfamilies, including those rich in glycine, rich in cysteine, rich in both cysteine and glycine, and those with low in cysteine and glycine [42]. In *Alligator mississippiensis*, investigations into the expression of *CBP* genes across different epidermal derivatives (e.g. claws, scales, and egg teeth etc.) and during different embryonic stages have unveiled unique spatial and temporal expression patterns of crocodilian *CBP* genes, highlighting the dynamic regulation of these genes during appendage development in *A. mississippiensis* [43]. Though the exact count of *CBP* genes in a tokay gecko *Gekko gecko* remains undetermined, at least 21 CBPs constitute the majority of the cornified material in the setae [15, 41], which are categorized based on their amino acid composition into glycine-rich, cysteine-rich, and serine-rich [44]. Immunohistochemical studies indicated that these distinct CBPs components are distributed at different layers of the setae, suggesting potential functional diversity [41]. The CBPs of lizard setae may be rich in cysteine or glycine. Glycine-rich CBPs, with its high hydrophobicity, can stabilize colloids in protoplasts and metabolic processes in tissues. Consequently, it can depress the freezing point and avert cell dehydration [45, 46]. In contrast, cysteine-rich CBPs, which containing sulfur atoms, can establish stable disulfide bonds, enhancing protein-protein interactions and endowing the setae with augmented mechanical strength. Moreover, cysteine is associated with the orientation of CBPs protein filaments in setae and the formation and branching of setae tips [15]. Cysteine-rich CBPs and glycine-rich CBPs confer a cornified layer that is both flexible and resistant. This is a feature that prevents damage to the epidermis during movement [11, 47]. Hence, delving into the amino acid composition of CBPs that constitute setae could be a crucial approach to studying the adhesive mechanism of setae.

A gecko *Gekko japonicus* is a pivotal species in the exploration of *CBP* genes, owing to its various epidermal derivatives, including harder claws and scales, as well as the highly elaborated adhesive setae [48]. A total of 72 *CBP* genes have been identified in the genome-wide identification analyses of *G. japonicus*, with the majority linked to the functional specialization of adhesive setae [33]. The expression profiles of certain *CBP* genes have shed further light on their indispensable role within the setae of *G. japonicus*. Investigations into the amino acid composition of CBPs in *G. japonicus* has indicated that setae are primarily composed of CBPs rich in specific amino acids, including glycine, proline, valine, serine, and leucine [44, 49]. It’s worth emphasizing that glycine-rich GjCBPs are instrumental during the maturation phase of setae [50]. The availability of chromosome-level genome data of *G. japonicus*, combined with transcriptomic analysis tools, presents an invaluable opportunity for delving into the expression dynamics of *CBP* genes in *G. japonicus*. As of now, 66 *GjCBP* genes have been identified in more precise chromosome level genome data [51]. However, thus far, it remains ambiguous as to whether GjCBPs of varied amino acid compositions manifest distinct expression profiles in setae [50].

During the embryonic development of the *G. japonicus*, the morphological characterization changes of the setae, as well as the underlying molecular mechanisms governed by *CBP* genes with varying amino acid compositions in setae maturation have yet to be elucidated. In this study, a morphological observation of the development of embryonic lamellae is conducted first. RNA sequencing (RNA-seq) is utilized to detect the relative expression levels of *GjCBP* genes at different stages of setae development. Quantitative real-time PCR (qRT-PCR) elucidates expression patterns of cysteine-rich and glycine-rich *GjCBP* genes in lamellar setae and dorsal scales, complemented by fluorescence in situ hybridization for specific *GjCBP* genes. This study will enrich the understanding of molecular mechanisms of *GjCBP* genes in *G. japonicus* setae development and pave the way for further exploring functions of GjCBPs within the setae.

## Results

### Embryo developmental stages and morphological features

**Fig. 1:**
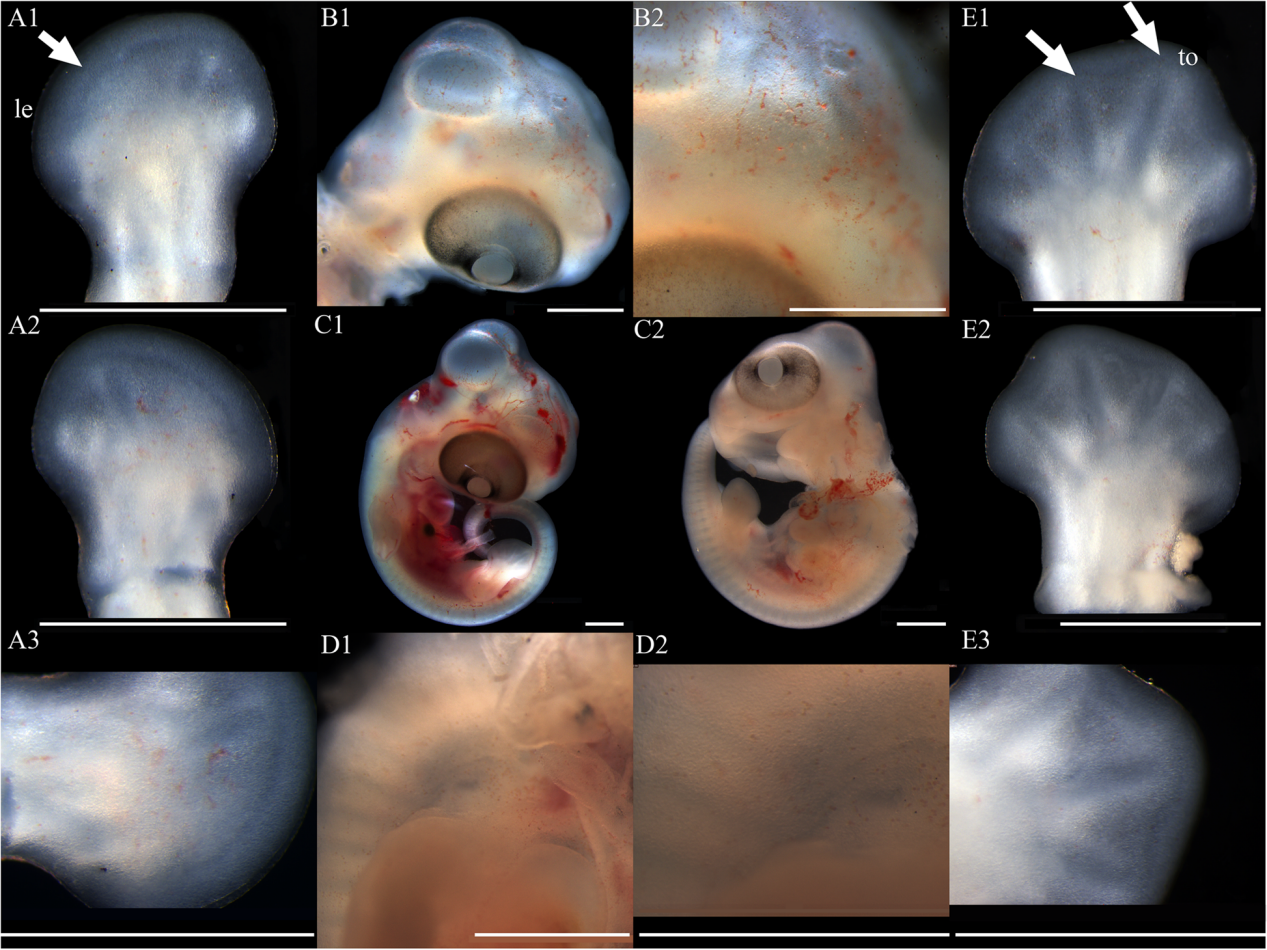
Development of *G. japonicus* embryos at stage 32 (Scale bar = 1 mm), *le* limb end, *to* toes. A1: dorsal surface of left forelimb; A2: ventral surface of left forelimb; A3: higher magnification view of the ventral surface of finger; B1: head; B2: higher magnification view of head; C1: right side of the whole embryo; C2: left side of the whole embryo; D1: back; D2: higher magnification view of back; E1: dorsal surface of right hindlimb; E2: ventral surface of right hindlimb; E3: higher magnification view of the ventral surface of toe

**Fig. 2:**
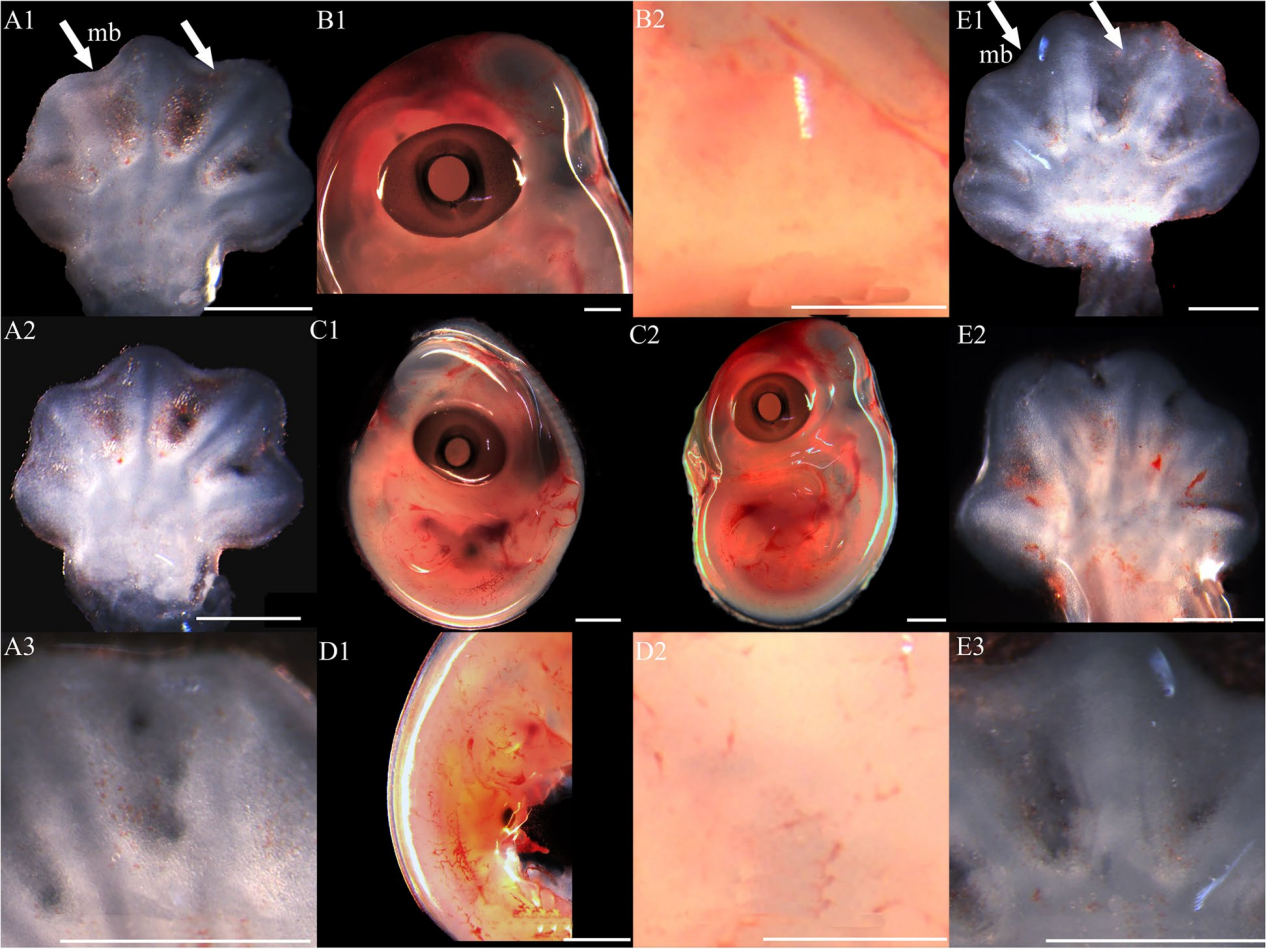
Development of *G. japonicus* embryos at stage 34 (Scale bar = 1 mm), *mb* membranous interdigital bands. For the description of the panels, refer to the caption of Fig. 1

**Fig. 3:**
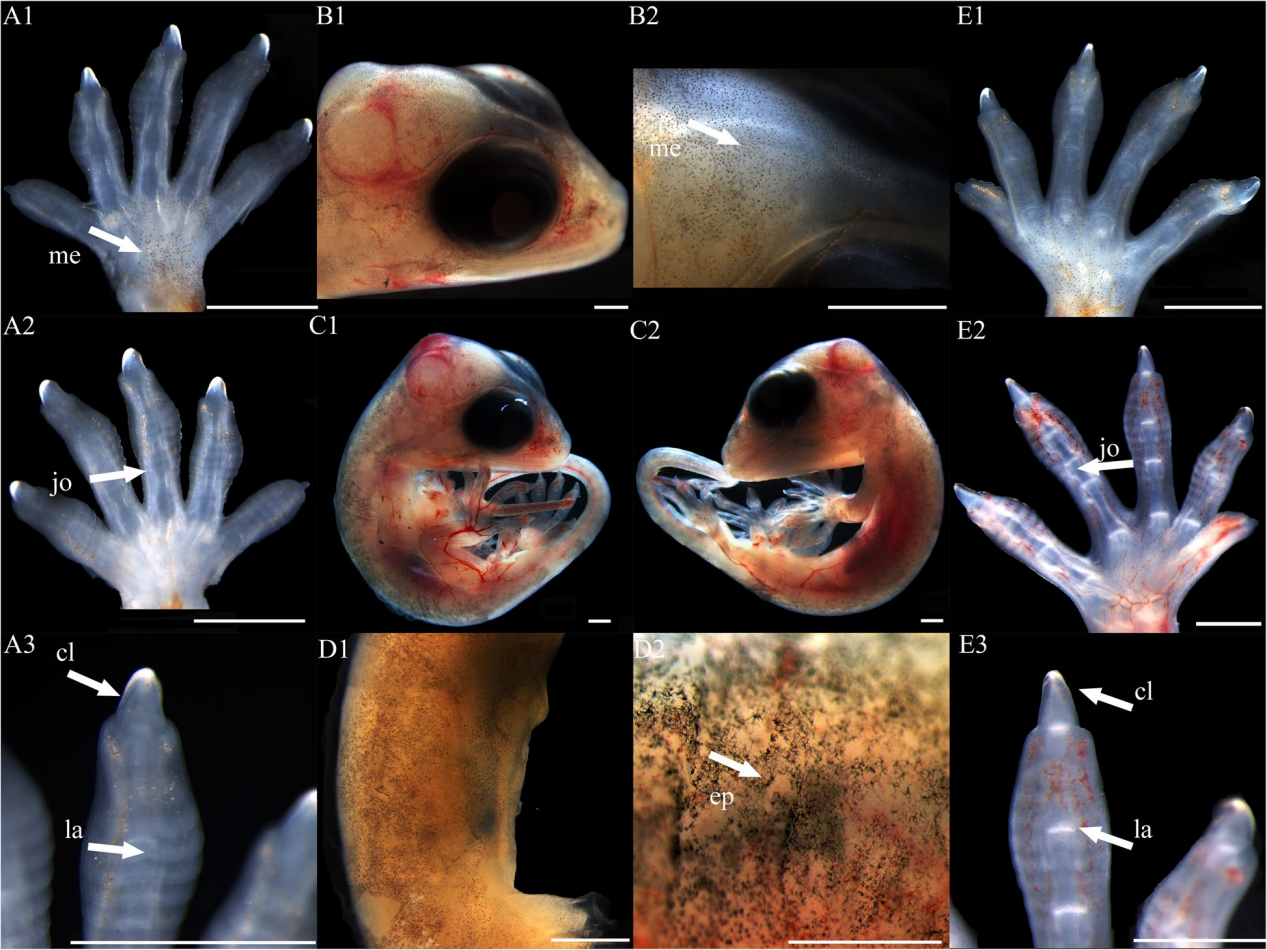
Development of *G. japonicus* embryos at stage 36 (Scale bar = 1 mm), *me* melanin, *ep* epidermis, *la* lamellae, *cl* claw, *jo* joint. For the description of the panels, refer to the caption of Fig. 1

**Fig. 4:**
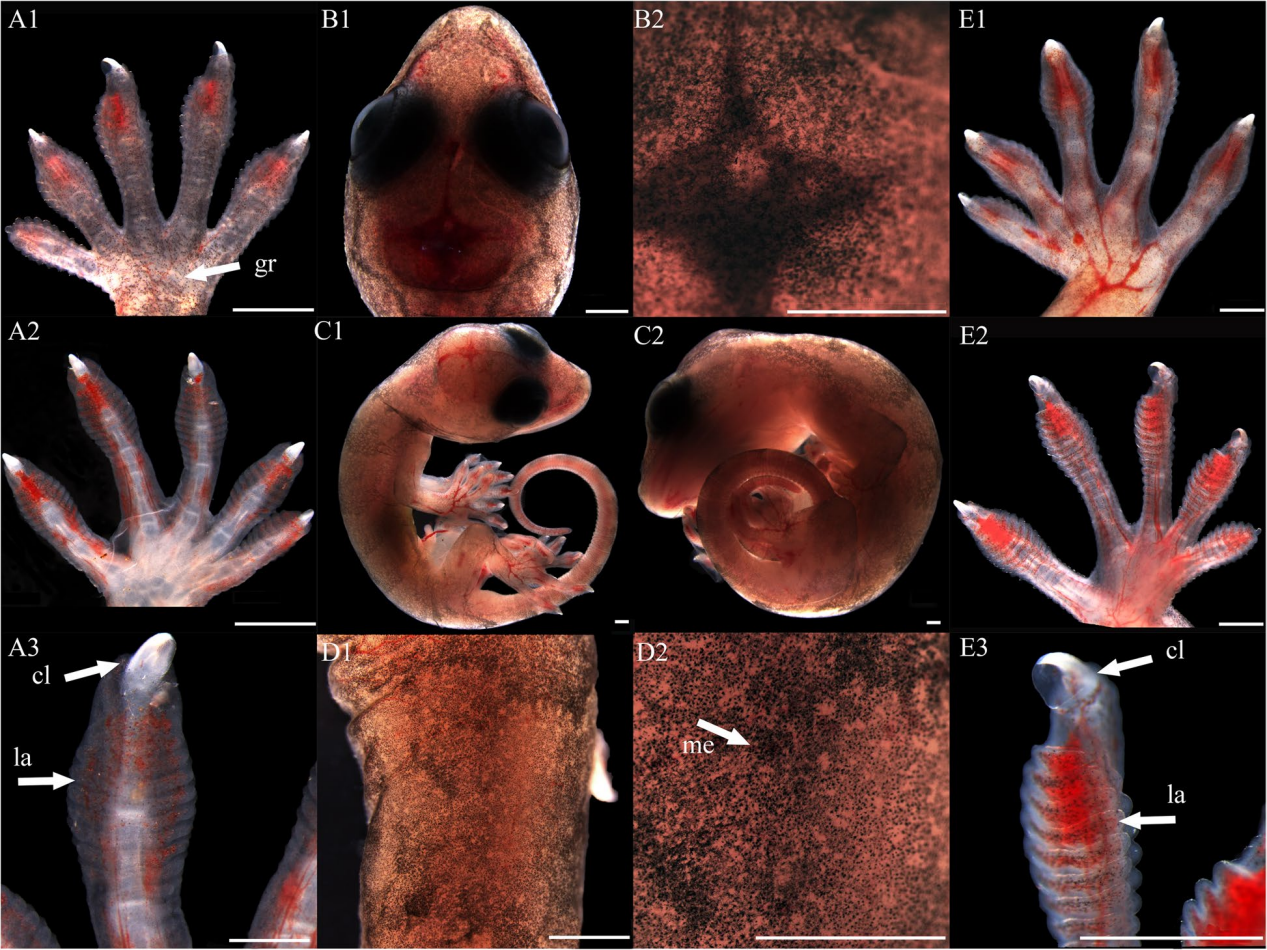
Development of *G. japonicus* embryos at stage 38 (Scale bar = 1 mm), *me* melanin, *gr* granulars, *cl* claw, *la* lamellae. For the description of the panels, refer to the caption of Fig. 1

**Fig. 5:**
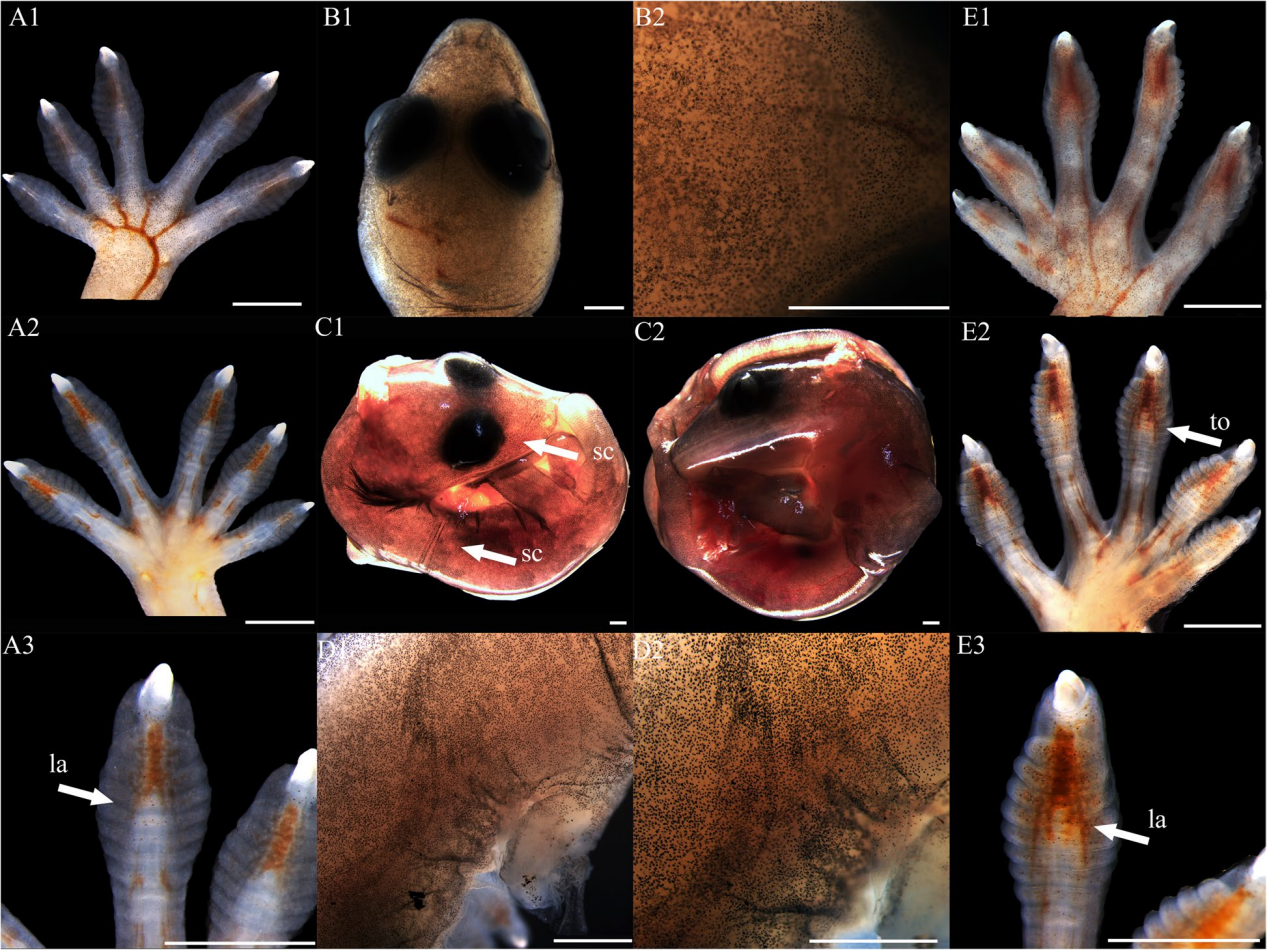
Development of *G. japonicus* embryos at stage 40 (Scale bar = 1 mm), *sc* scales, *to* toes, *la* lamellae. For the description of the panels, refer to the caption of Fig. 1

**Fig. 6:**
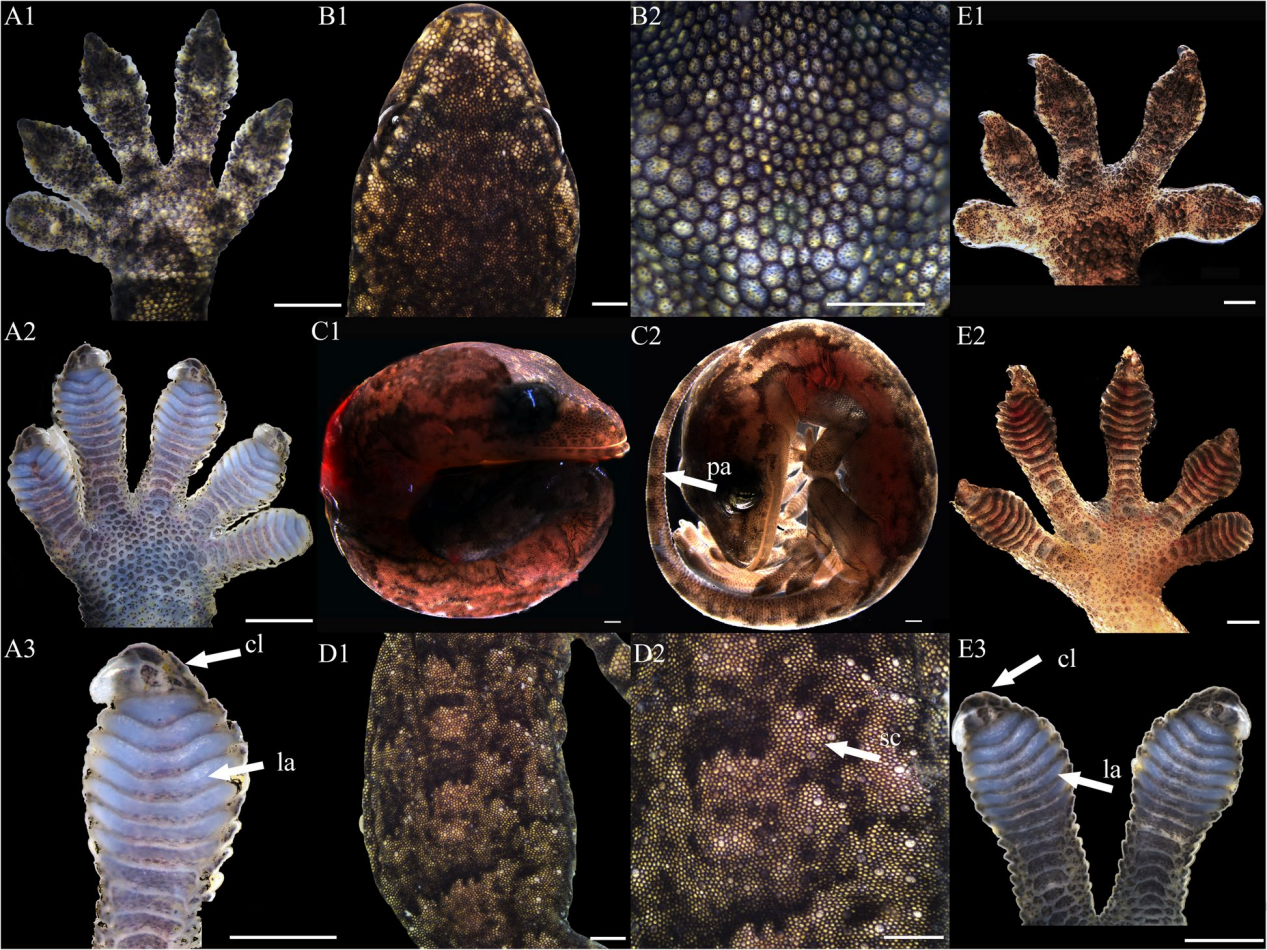
Development of *G. japonicus* embryos at stage 42 (Scale bar = 1 mm), *sc* scales, *to* toes, *la* lamellae, *p.a*. patterns on the tail. For the description of the panels, refer to the caption of Fig. 1

At stage 32, the entire body surface is smooth with no pigmentation or epidermis visible. The limbs, limb columns, and limb rods begin to take shape, with the limb ends enlarging (Fig. 1A1, A2, A3, E1, E2 and E3). Some specimens reveal discernible toes III and IV on the hind limbs, whereas other toes remain elusive. The toes are smooth with no cornified features (Fig. 1E1). At stage 34, the entire embryo retains smoothness with no melanin deposition, and the epidermis remains invisible. Both forelimbs and hindlimbs, being semi-transparent, display discernible digits and toes at their extremities, connected by curved membranous interdigital bands, which are noticeably thicker than the interdigital bands, with slight indentations between digits (Fig. 2A1 and E1). No cornified features are present. At stage 36, melanin becomes prominently visible across the embryo surface (Fig. 3A1 and B2), and the emergence of the epidermis begins (Fig. 3D2). Toes elongate, and claws start to form, which is noticeable despite their transparency and lack of clear differentiation. The ventral surface of the digits shows lamellae outlines, albeit not yet fully formed (Fig. 3A3 and E3). Annular bones become apparent on the phalanges, indicating the progress of skeletal and joint development (Fig. 3A2 and E2). The cornified features remain absent. At stage 38, melanin deposition on the embryo surface further intensifies, accompanied by the further maturation of body epidermis, albeit devoid of visible scales (Fig. 4D2). In contrast, limbs exhibit faint outline of granulars (Fig. 4A1). The hind limbs outstretch the forelimbs in length, and most specimens present fully developed robust claws at the end of digits and toes (Fig. 4A3 and E3). Concurrently, the digits and toes undergo further elongation. The phalanges and the semitransparent pad lamellae become more defined, and a slight melanin deposition is noted (Fig. 4A3 and E3). At stage 40, the presence of dorsal tubercles, labial scales, chin scales, circumnasal scales, and granulars on the body is clear, albeit not fully developed. The dorsal granulars appear faintly, and the limbs exhibit visible scales. Despite the overall light color, the patterns on the back and tail become discernible as the body assumes full coloration (Fig. 5C1). Concurrently, the limbs darken, and the toes undergo further development, while retaining a semi-transparent state (Fig. 5E2). Most specimens show further development of pad lamellae, characterized by increased melanin deposition (Fig. 5A3 and E3). At stage 42, the body color exhibits a significant deviation from stage 40, the body dorsal surface becomes entirely covered with visible scales. The skin color deepens to a uniform gray-brown and assumes complete opacity (Fig. 6D2). Patterns on the back and tail become clear (Fig. 6C2), with the ventral color presenting a lighter, flesh-colored tone. The hind limbs outstretch the forelimbs in length. The pad lamellae attain full development. Claws become fully differentiated and visible (Fig. 6A3 and E3); embryos of this stage resemble newly hatched juveniles, already have climbing ability.

### Assembly and information analyses of transcriptome data

To investigate the expression levels of *CBP* genes in the setae of *G. japonicus*, transcriptome sequencing was conducted on total RNA, extracted from the pad lamellae sample of embryos at stages 32, 34, 36, 38, 40, and 42. Each stage sample comprising three biological replicates. The cDNA libraries were sequenced on the Illumina Novaseq6000 platform. The total raw reads for all 18 samples ranged from 43,543,010 to 87,176,000 (Table S3). Following quality control, which involved the removal of adapters and low-quality bases (quality score < 20), the total clean reads for all samples ranged from 43,302,140 to 86,093,358, and the total clean bases ranged from 6,504,703,620 bp to 12,928,576,617 bp. The ratio of clean reads to raw reads for the 18 libraries fluctuated between 98.43% and 99.45%, while the proportion of Q30 bases (nucleotides with a quality value ≥ 30) lay between 93.55% and 94.50%.

The clean reads were mapped to the *G. japonicus* reference genome database using HISAT, yielding total mapped reads ranged from 41,055,029 to 81,110,267 for all samples, with mapping rates between 92.30% and 94.80% (Table S4). A total of 8977 unique genes were generated from the clean reads (Table S5), which were subsequently mapped with the *G. japonicus* reference genome database using StringTie. The Pearson correlation coefficient was calculated to reflect the gene expression correlation between the control and treatment group samples (Fig. S2). Overall, these data validate the accuracy of the classification into the six stages and pave the way for further exploration of the distinct genes identified.

### Expression pattern of the *GjCBP* genes from pad lamellae of *G. japonicus* embryos

**Fig. 7:**
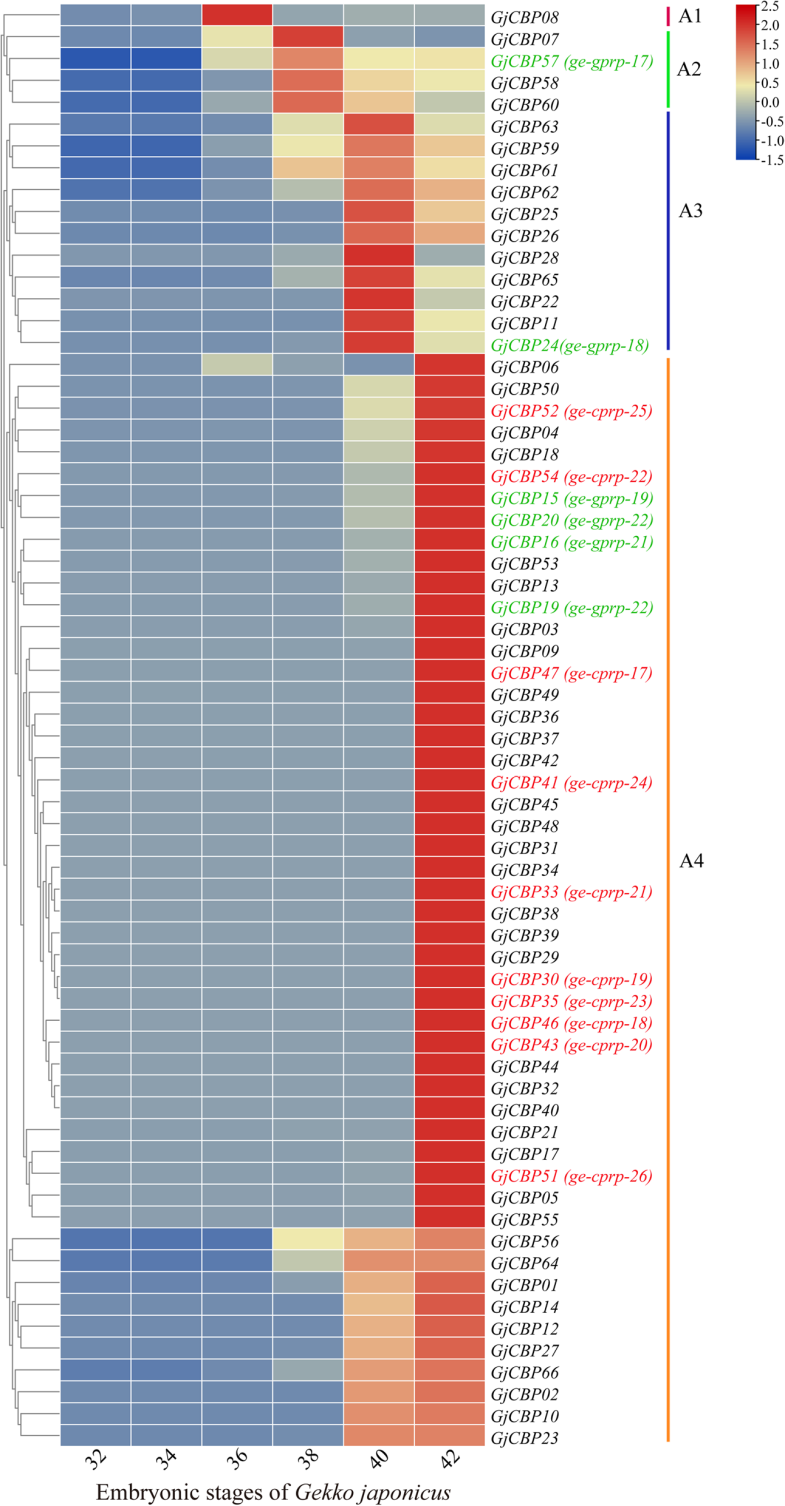
Expression patterns of *GjCBP* genes during development of pad lamellae from *G. japonicus* embryos. The color scale represents TPM which were normalized log10 transformed counts. -1.5 to 2.5 was artificially set with the color scale limits according to the normalized value. The red color of the bar represents higher expression. The genes in red font represent the cysteine-rich CBP-encoding genes studied in qRT-PCR, the genes in green font represent the glycine-rich CBP-encoding genes studied in qRT-PCR, and A1, A2, A3, A4 represent the four clusters of genes with different expression patterns, respectively

To validate the involvement of unigenes in the developmental stages of *G. japonicus*, the TPM values of the unigenes were utilized to represent the expression levels of genes. The expression patterns of *GjCBP* genes in *G. japonicus* during various embryonic stages were investigated. All 66 *GjCBP* genes involved in the study were found to express during setae development (Fig. 7, Table S6), with most *CBPs* genes reaching their highest expression levels at stages 40 and 42. Based on gene expression patterns, they can be divided into four clusters: Cluster A1 consists of a single *GjCBP* genes, with its relative expression level peaking at stage 36; Cluster A2 consists of four *GjCBP* genes, with their relative expression levels peaking at stage 38; Cluster A3 consists of 11 *GjCBP* genes, with their relative expression levels peaking at stage 40; Cluster A4 consists of the majority of *GjCBP* genes, with their relative expression levels peaking at stage 42. These patterns indicate that a small portion of genes have higher expression levels at stages 36 to 40. Except for the glycine-rich CBP encoding genes *ge-gprp-17* and *ge-gprp-18*, the other haphazardly selected glycine-rich CBP encoding genes and cysteine-rich CBP encoding genes showed similar expression patterns during the development of setae.

### Analysis of differential expressed genes in different developmental stages of pad lamellae

The six stages were employed for pairwise comparisons through nine comparison groups, including stages 34 versus 32, 36 versus 32, 38 versus 32, 40 versus 32, 42 versus 32, 36 versus 34, 38 versus 36, 40 versus 38 and 42 versus 40. These genes were differentially expressed among specific developmental stages, as shown in volcano plot (Fig. S3).

### *GjCBP* genes expression profiles of glycine-rich CBP encoding genes and cysteine-rich CBP encoding genes in pad lamellae and dorsal scales of *G. japonicus* embryonic stages

**Fig. 8:**
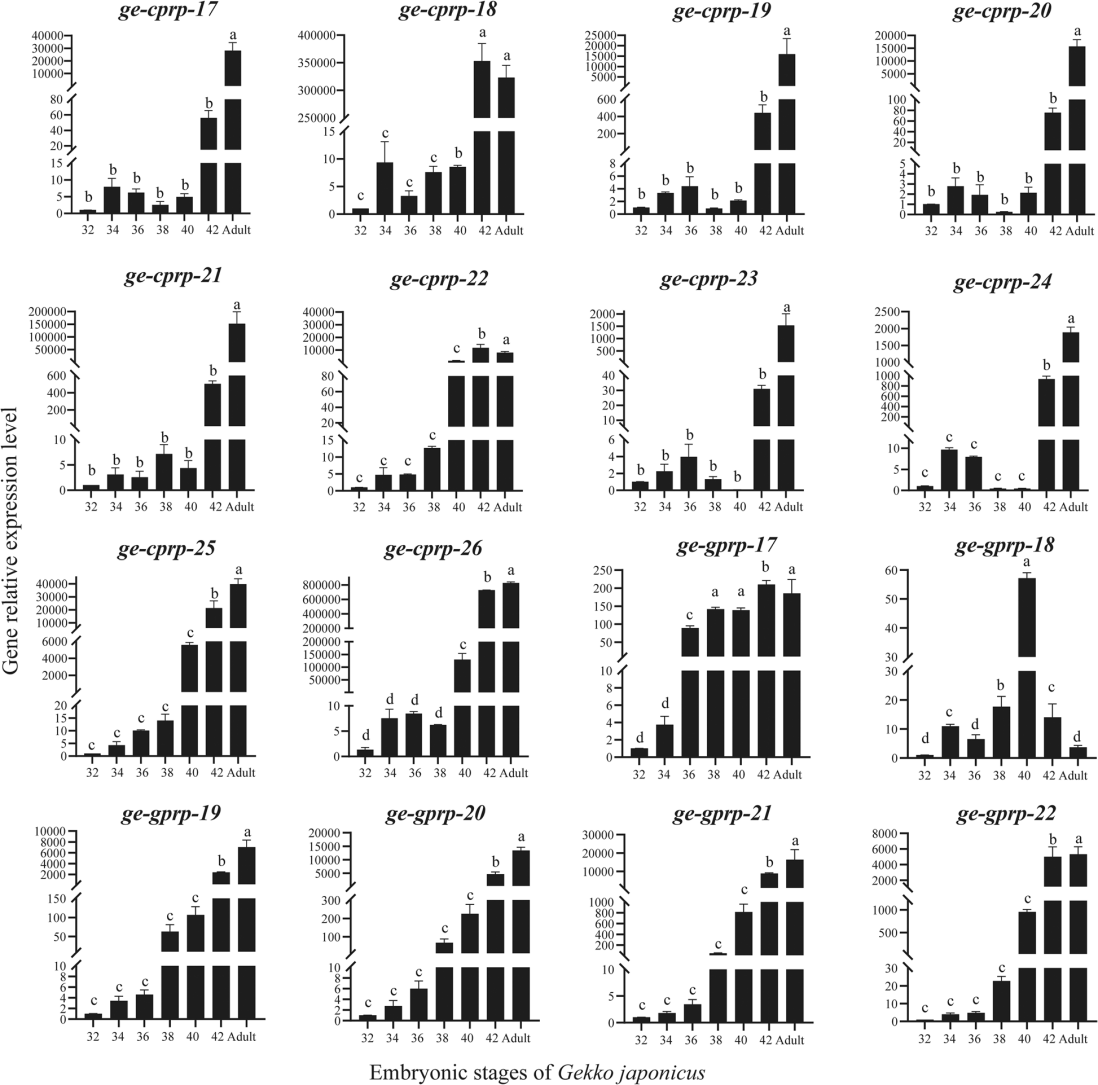
Relative gene expression levels of *ge-cprp-17* ~ ge-cprp-26 and ge-gprp-17 ~ ge-gprp-22 in pad lamellae from embryonic stages (32–42) and adult of *G. japonicus*. The *EF-1α* gene of *G. japonicus* (GenBank Accession No. AF199487) serving as the reference gene. The horizontal axis represents embryonic developmental stages 32, 34, 36, 38, 40, 42 and adult, and the verticalaxis represents the relative expression with the expression level at stage 32 as a reference. Different letters represent significant differences (*p* < 0.05) using ANOVA test among samples. Error bars indicate standard deviation of three independent replicates

**Fig. 9:**
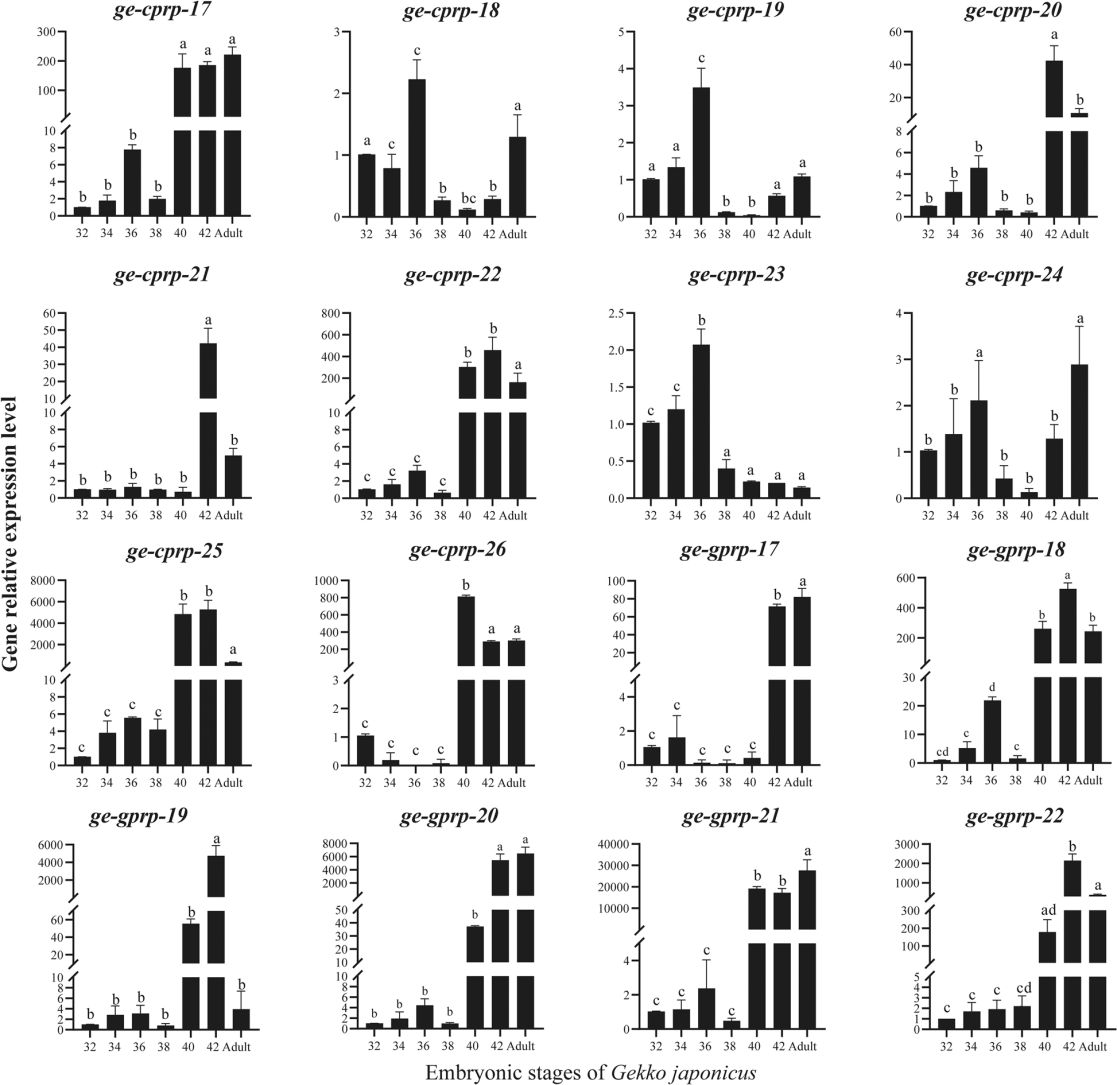
Relative gene expression levels of *ge-cprp-17~ge-cprp-26* and *ge-gprp-17~ge-gprp-22* in the dorsal epidermis from embryonic stages (32-42) and adult of *G. japonicus*. The *EF-1α* gene of *G. japonicus* (GenBank Accession No. AF199487) serving as the reference gene.The horizontal axis represents embryonic developmental stages 32, 34, 36, 38, 40, 42 and adult, and the verticalaxis represents the relative expression with the expression level at stage 32 as a reference. Different letters represent significant differences (*p* < 0.05) using ANOVA test among samples. Error bars indicate standard deviation of three independent replicates

The expression patterns of *ge-cprp-17* ~ *ge-cprp-26* and *ge-gprp-17* ~ *ge-gprp-22* genes were further probed in pad lamellae (Fig. 8) and dorsal epidermis (Fig. 9) using qRT-PCR technique. All these genes expressed during all six embryonic developmental stages and in adults, with variations in expression levels at different stages. The expression patterns of *ge-cprp-17 ~ ge-cprp-26 and ge-gprp-17 ~ ge-gprp-22* genes in setae were similar, they were significantly upregulated at stage 40 or 42, reaching the highest expression levels during embryonic development. This concurs with the transcriptome sequencing results. Besides, most of the genes also have high relative expression levels in adult. However, the relative expression levels of these genes in the dorsal epidermis varied. The *ge-cprp-17*, *ge-cprp-22*, *ge-cprp-25*, and *ge-cprp-26* genes had lower expression from stages 32 to 38, then significantly up-regulated at stages 40 or 42 (Fig. 9*ge-cprp-17*, *ge-cprp-22*, *ge-cprp-25* and *ge-cprp-26*). The *ge-cprp-18*, *ge-cprp-19*, *ge-cprp-20*, *ge-cprp-21*, and *ge-cprp-22* genes exhibited relatively low expression throughout the development of dorsal epidermis without any obvious trend (Fig. 9*ge-cprp-17*, *ge-cprp-22*, *ge-cprp-25* and *ge-cprp-26*). The majority of glycine-rich CBPs encoding genes showed relatively low expression levels during stages 32 to 38 of embryonic development, and significantly increased at stage 40 or 42, displaying relatively high expression levels (Fig. 9*ge-gprp-17 ~ ge-gprp-22*). It is remarkable that the relative expression levels of different *GjCBP* genes differed considerably between stage 40 and 42. For instance, although *ge-cprp-21* and *ge-cprp-23* have similar expression patterns in pad lamellae, there is a significant gap in their relative expression levels at stages 40 and 42.

### *In situ* hybridization of cysteine-rich CBP encoding gene *(ge-cprp-17)* and glycine-rich CBP encoding gene (*ge-gprp-19*) in the pad lamellae and dorsal scales of *G. japonicus* embryonic stages 40 and 42

**Fig. 10:**
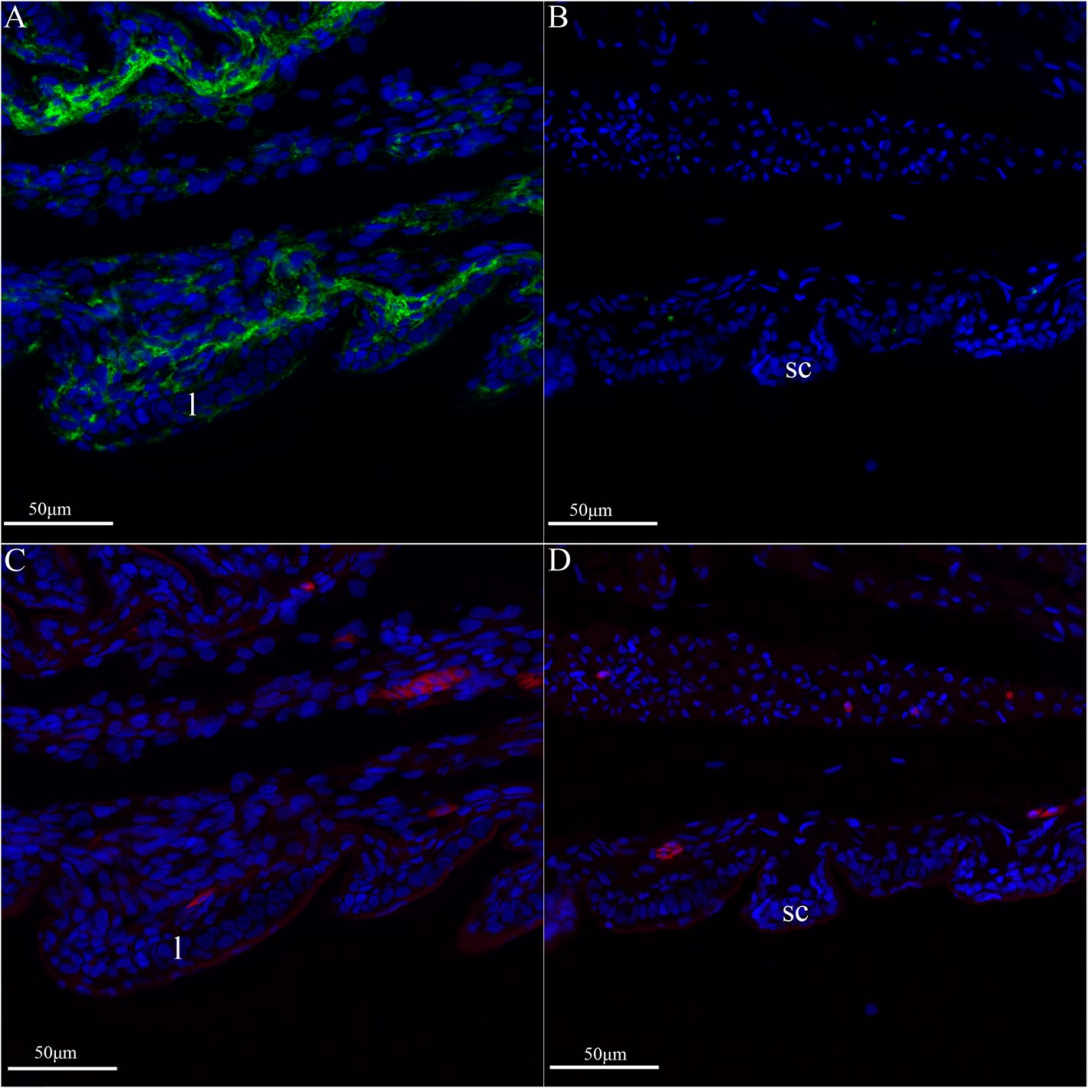
*In situ* hybridization results of* ge-gprp-19* (**A**, **B**) and *ge-cprp-17* (**C**, **D**) of *G. japonicus* at the right rear toe lamellae (**A**, **C**) and dorsal epidermis (**B**, **D**) of embryonic stage 40.The red fluorescent signal is *ge-cprp-17*, green fluorescent signal indicates *ge-gprp-19*, and blue is nuclear staining. *l* lamellae, *sc* scales

The spatiotemporal expression patterns of the *ge-cprp-17* and *ge-gprp-19* were assayed in right rear toe lamellae and in the dorsal epidermis (Fig. 9) using in situ hybridization. In addition, the development of right rear toe lamellae, setae and scales was investigated, as well as the differentiation of epidermal cells. At stage 40, the right rear toe lamellae have shown up, but not yet the setae. Dorsal scales are developing. The nuclear markers (blue) indicate that differentiation of the epidermal cell is not yet apparent (Fig. 10A and C). The glycine-rich CBP encoding gene, *ge-gprp-19* (green), manifested pronounced hybridization fluorescence signals in right rear toe lamellae (Fig. 10A), while almost no fluorescence was detected in the dorsal scales (Fig. 10B). On the other hand, the cysteine-rich CBP encoding gene, *ge-cprp-17* (red), revealed moderate fluorescence signals in both the right rear toe lamellae and dorsal scales (Fig. 10C and D). This indicates that, in the stage 40, *ge-gprp-19* gene is predominantly expressed in the right rear toe lamellae but scarcely expressed in the dorsal scales, while *ge-cprp-17* exhibits relatively low expression in both the lamellae and dorsal scales.

**Fig. 11:**
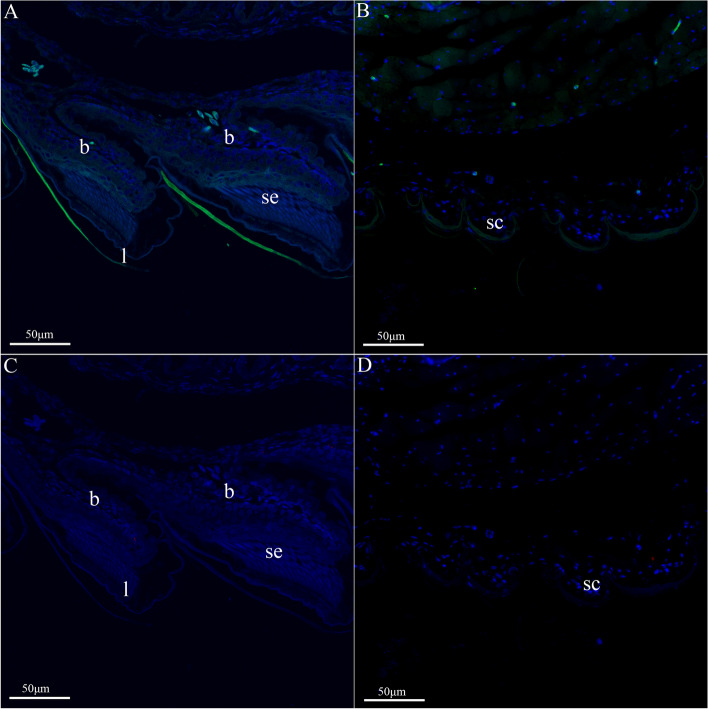
*In situ* hybridization results of* ge-gprp-19* (**A**, **B**) and *ge-cprp-17* (**C**, **D**) of *G. japonicus* at the right rear toe lamellae (**A**, **C**) and dorsal epidermis (**B**, **D**) of embryonic stage 42. The red fluorescent signal is *ge-cprp-17*, green fluorescent signal indicates *ge-gprp-19*, and blue is nuclear staining. *l *lamellae, *sc* scales, *se* setae, *β*(b) beta layer cell

At stage 42, setae have formed on the right rear toe lamellae and epidermal cell differentiation is more obvious, with clearly differentiated beta layer cells visible (Fig. 11). Dorsal scales are fully developed. *ge-gprp-19* (green) showed strong signals in right rear toe lamellae, whereas a faint fluorescence was noted in the dorsal skin (Fig. 11). This implies that *ge-gprp-19* continues to be highly expressed in right rear toe lamellae and low in dorsal skin at stage 42. Distinct setae observed at this stage confirm their formation. On the other hand, neither the right rear toe lamellae nor the dorsal scales, devoid of any red fluorescence detected, indicating that the expression of *ge-cprp-19* in the dorsal scales is likely minimal at this developmental stage.

## Discussion

Setae, fundamental cornified structures in *G. japonicus*, positioned on the pad lamellae, enabling the geckos to traverse smooth surfaces. CBPs, pivotal to the composition of setae, and the amino acid composition of GjCBPs may be a potential determinant of their adhesive function.

The transcriptomic data demonstrates that all 66 *GjCBP* genes play roles in the maturation of lamellar setae (Fig. 7), In *Gallus gallus*, RNA-seq analyses indicated that all *CBP* genes are involved in feather development, with significant variations in the expression levels of different *CBP* genes across various stages of feather maturation [52]. Analyses based on RNA samples from the carapace, limbs, neck, and tail of two turtle species showed that the majority of *CBP* genes could be successfully amplified [53, 54]. Conclusively, all *GjCBP* genes participate in the formation of setae, and the embryonic developmental stages from 40 to 42 are the key periods for lamellar setae formation in *G. japonicus*.

The qRT-PCR detection revealed that during the embryonic development at stages 40 and 42, as well as in adults, glycine-rich GjCBP encoding genes manifest high expression levels in both dorsal epidermis and lamellae setae. Predominantly, the majority of the cysteine-rich GjCBPs encoding genes manifest elevated relative expression levels in the lamellar setae, with subdued levels in the dorsal epidermis. The majority of *GjCBP* genes exhibit relatively high expression levels in adults (Figs. 8 and 9), geckos may lose some setae and lipids on the substrate as they climb [55, 56], and shed epidermal derivatives continuously as they grow. The setae of *G. japonicus* may also need to be constantly renewed and replenished to maintain efficient adhesion [57, 58]. In tokay gecko, hydrophobic CBPs, notably glycine-rich ones like GE-GPRP-6 and HgG5-positive proteins prevalent in most lizards [59], are positioned in the β-layer beneath the cornified stratum of the setae [32]. Delving into the expression patterns of *GjCBP* genes in *G. japonicus* setae highlights the instrumental role of glycine-rich GjCBPs encoding genes in setae formation [50]. Thus, it’s plausible to infer that glycine-rich GjCBPs might serve functions analogous to those in other reptiles, consistently being situated in the resilient β-layer to counteract cellular dehydration. This might elucidate their heightened expression in dorsal scales and lamellar setae during stages 40, 42, and in adults. Additionally, gecko setae primarily comprise CBPs enriched in serine and tyrosine, cysteine-rich CBPs, alpha keratins, lipids, and lipoproteins. The quantity of cysteine-rich CBPs appears intrinsically linked to the compositional makeup of the setae, integral to the adhesive pads [15, 44]. Cysteine-rich CBPs might perform protective roles in setae, such as preventing water loss and UV damage. In mammals, cysteine-rich keratins predominantly manifest in epidermal structures, including hair, nails, and the keratin layer, and are intrinsically linked to tissue hardness and tensile strength [60, 61]. Immunolocalization studies in geckos also confirmed the essential roles of glycine-rich CBPs and cysteine-rich CBPs in setae [32, 59]. Comparative investigations contrasting hard-shelled and soft-shelled turtles, revealed that CBP with β-sheets containing proline and cysteine might not be conducive to formation of hard corneous material [53]. In *Alligator sinensis*, a blend of immunohistochemical and biochemical analysis indicated the presence of both cysteine-rich CBPs and glycine-rich CBPs in the transitional and corneous layer of the claw. The pronounced cysteine content in these proteins suggests potent cross-linking capabilities, culminating in a highly rigid corneous layer [35, 62, 63]. Turtle claws primarily consist of CBPs that are notably basic and rich in cysteine. These CBPs accumulate in the upper and transitional layers of the claw epidermis, establishing intricate crosslinking networks, which in turn amplify the mechanical resistance of the claws [35]. In *Gecko gecko*, CBPs rich in glycine, cysteine, and serine were detected and are located in different parts of the setae, they are all essential proteins constituting the setae [44, 64]. Hence, we postulate that glycine-rich GjCBPs might be instrumental in both dorsal scales and lamellar setae, potentially augmenting the mechanical integrity of these structures. Additionally, cysteine-rich GjCBPs may also be an essential component of *G. japonicus* setae, potentially serving as specialized structural proteins that amplify the flexibility of setae. This insight could be pivotal in exploring the nexus between the amino acid composition of CBPs and the intrinsic attributes of setae.

However, even for GjCBP encoding genes with similar amino acid fractions, their relative expression levels may vary considerably in the same epidermal derivative, as is the case for *ge-cprp-21* and *ge-cprp-23* at stages 40 and 42 of setae development (Fig. 8). Morphological findings suggest that the pad lamellae have been identified at stage 40 (Fig. 5), and is basically well developed at stage 42 (Fig. 6). This suggests that different *GjCBP* genes have different regulatory functions at the stage of pad lamellae formation, and thus the relative expression levels of different *GjCBP* genes are widely different. Similar findings were reported in study on expression patterns of *GjCBP* genes in the overall embryonic development and lamella of *G. japonicus* [50, 65], and we speculate that even GjCBPs with similar amino acid compositions may play diverse functions. This provides new insights for us to subsequently explore the function of GjCBPs.

Fluorescence in situ hybridization analyses reveal that *ge-gprp-19* is prominently expressed in pad lamellae at stages 40 and 42. Yet, its expression is undetectable in dorsal scales at stage 40, with only marginal expression was found at stage 42. In contrast, *ge-cprp-17* manifests pronounced expression in lamellar setae and dorsal scales at stage 40. However, the expression of the gene was not detected in neither in lamellae nor in dorsal scales at stage 42 (Figs. 10 and 11). Delving into the expression patterns of *CBP* genes during the feather maturation process in chickens via in situ hybridization, it becomes evident that CBPs genes manifest varied expression patterns across different feather types [52]. Immunohistochemical studies on the lamellae of the *A. carolinensis* indicate that CBPs rich in glycine, CBPs rich in cysteine, and CBPs rich both glycine and cysteine exhibit different distribution patterns across distinct setae regions [59]. Moreover, in *G. gecko*, it was found that CBPs, differentiated by their amino acid compositions, localize in distinct setae regions, potentially fulfilling diverse roles [32]. Consequently, it’s plausible to infer that cysteine-rich CBPs encoding genes and glycine-rich CBPs genes encoding have spatial-temporal expression pattern differences in the development of epidermal scales and/or lamellar setae. Prior work has demonstrated the crucial role of glycine-rich CBP encoding genes in setae development [50], whereas the present study demonstrated that both glycine-rich CBP encoding genes and cysteine rich CBP encoding genes contribute to setae formation, with distinct spatiotemporal and temporal expression patterns, as evidenced by the transcriptome, qRT-PCR, and in situ hybridization.

Transcriptome and qRT-PCR results indicated that the relative expression levels of most genes were significantly higher at embryonic developmental stages 40 or 42 than at other stages. Previous inquiries into the expression patterns of crocodilian *CBP* genes have shown that embryonic stages and/or tissue types influence the relative expression levels of different *CBP* genes [43]. Studies on the expression patterns of *GjCBP* genes in *G. japonicus* found that the relative expression levels of *GjCBP* genes were higher in stages 39 and 42 [50]. Morphological results of this study also indicated that at stage 40 only the developing pad lamellae was present (Fig. 5), whereas at stage 42 the lamellae was fully developed and the individuals already had climbing ability (Fig. 6), which suggests that the setae were fully developed at stage 42. In situ hybridization showed that no cell differentiation occurred in right rear toe lamellae at stage 40, whereas newly differentiated beta layer cells were clearly visible in right rear toe lamellae at stage 42, and setae were embedded in the lamellae. Setae growth was believed to be closely related to beta layer differentiation, and beta cells located above the basal layer would form bundles of CBPs filaments and gradually move upward [65]. Stages 40 to 42 may be a critical stage in the development of the setae. However, previous studies have shown that complete lamellae are present at stage 39 of *G. japonicus* and that the glycine rich GjCBP encoding genes has a high relative expression level. We suggest that material accumulation begins at stage 39 in embryos, followed by differentiation of the various cellular layers, and ultimately the appearance of setae at stage 42. Stages 39 to 42 are critical periods of setae development in *G. japonicus*. Future studies on the development of setae in *G. japonicus* should focus on stages 39 to 42.

In future, downregulating or knock-down the expression or overexpression of cysteine-rich genes, among other setae-associated genes, might shed light on the specific roles of *GjCBP* genes within setae [66], thereby gaining a more comprehensive understanding of the molecular regulatory mechanisms of their cornification/keratinization process. Additionally, epidermal derivatives of *G. japonicus* may result from the joint accumulation of GjKRTs (*G. japonicus* alpha keratins) and GjCBPs [65]. Future exploration of epidermal derivatives should also focus on the interaction between GjKRTs and GjCBPs.

## Conclusion

Setae, as an interesting cornified material, have garnered widespread attention due to their unique characteristics. These structures enable certain reptiles to effortlessly traverse smooth vertical surfaces. Most importantly, CBPs stands out as a crucial component of these setae, playing a pivotal role in both their function and structure.

Through morphological observations, transcriptome sequencing, qRT-PCR, and fluorescence in situ hybridization, this study investigated the morphological changes in pad lamellae during the embryonic development of the *G. japonicus*, as well as the molecular mechanisms of glycine-rich CBP encoding genes and cysteine-rich CBP encoding genes in the development process of the setae. All 66 CBPs were found to be instrumental in setae formation. Glycine-rich CBPs play a crucial role in the development of dorsal scales and setae, while the majority of cysteine-rich CBPs seem to be essential components of *G. japonicus* setae. However, even GjCBPs with similar amino acid compositions may play diverse functions. Additionally, distinct spatiotemporal expression patterns between the glycine-rich and cysteine-rich CBP encoding genes during epidermal scale and/or setae formation were observed. Stages 39 to 42 are critical periods of setae development in *G. japonicus*. These findings not only advance our understanding of the molecular mechanisms but also provide a foundation for further investigation into the functions of GjCBPs in the development of *G. japonicus* setae. Furthermore, given the potential applications of mimicking gecko adhesion in biomedical and industrial contexts, understanding these intricacies holds promise for the design of innovative adhesive materials. Future studies should delve deeper into the specific roles of various GjCBPs, and the potential interactions between GjCBPs and GjKRTs during the cornification/keratinization process, thereby further illuminating the multifaceted world of *G. japonicus* adhesion.

## Materials and methods

### *G. japonicus* culturing, sample collection and morphological observation

In this study, the relevant procedures were approved by the Institutional Animal Care and Use Committee of Nanjing Normal University [SYXK (Jiangsu) 2020–0047 and IACUC-20220258]. From May to July 2022, a total of 60 *G. japonicus* were collected from several locations in Nanjing (32°03′N, 118°45′E), eastern China. These geckos were subsequently housed at the Herpetological Research Center of Nanjing Normal University in customized mesh cages, with a steady environmental temperature of 28 °C and were predominantly fed during the night [67]. The cages were organized to maintain a male-to-female ratio of 2:1, with a maximum of 15 individuals per cage [68]. Additionally, pregnant female geckos were individually situated in semi-transparent small rearing boxes (500 mm × 300 mm × 250 mm), which were lined with plastic wrap to ensure the integrity of the eggs and facilitate collection, with the environmental temperature maintained at 28 °C. A hatching substrate for the eggs was prepared using perlite and water in a 1:1 mass ratio. The eggs were meticulously collected and placed in boxes containing the hatching substrate, and then kept in an incubator at 28 °C, with the humidity of the perlite substrate controlled at approximately − 12 kPa. A total of 38 intact fertilized embryos were finally collected, the use of all experimental *G. japonicus* in this paper is described in Table S1. We selected embryos from six stages of development, specifically, stage 32 (10–13 days), stage 34 (13–19 days), stage 36 (22–28 days), stage 38 (30–35 days), stage 40 (36–43 days), and stage 42 (46–56 days), for morphological observations. Three biological replicate samples were collected for each development stage for a total of 18 embryos. 1×PBS buffer (PH = 7.2) was used to provide a stable liquid environment for embryonic observation. The development characteristics of the embryos at the six stages were scrutinized under Nikon SMZ1500 dissection microscope. We focused on the following morphological features of the embryo, including: Dorsal surface of left forelimb; Ventral surface of left forelimb; Higher magnification view of the ventral surface of finger; Head; Higher magnification view of head; Right side of the whole embryo; Left side of the whole embryo; Back; Higher magnification view of back; Dorsal surface of right hindlimb; Ventral surface of right hindlimb; Higher magnification view of the ventral surface of toe; Finally, photoshop was used to do the necessary processing and stitching of the images. After recording the morphological characteristics, the samples were preserved in 4% paraformaldehyde.

### RNA isolation, library construction, and sequencing

To elucidate the expression profiles of *GjCBP* genes during the development of embryonic setae, total RNA was extracted from the dorsal epidermis and pad lamellae of embryos at stages 32, 34, 36, 38, 40, 42 and adult. Each stage comprised three biological replicates, leading to a total of 18 embryos and 3 adult samples. We euthanized geckos by freezing them to -20 °C and then collected tissue samples immediately for further analysis. The geckos was taken under a Nikon SMZ1500 dissecting microscope, and a small piece of the dorsal epidermis and all the pad lamellae were carefully dissected with a scalpel and forceps, quickly deposited in liquid nitrogen for RNA extraction, and the remainder placed in liquid nitrogen and stored at -80 °C. The TransZol Up Plus RNA Kit (TransGen Biotech, Beijing, China) was employed for RNA extraction, adhering to the standard protocol. The NanoDrop 2000 (Thermo Scientific, Waltham, USA) was utilized to assess the concentration and quality of the purified total RNA samples, measuring the absorbance ratios at 260–280 nm (A260/A280) and 260 nm to 230 nm (A260/A230). RNA integrity was confirmed via 1.0% agarose gel electrophoresis utilizing the Agilent 2100 system. Reverse transcription was performed with HiScript II Reverse Transcriptase (Vazyme, Nanjing, China) using 1000 ng of total RNA meeting the quality criteria (A260/A280 > 1.8 and A260/A230 > 2.0), in accordance with the manufacturer’s instructions. The resultant cDNA samples were preserved at -20 °C for subsequent RNA-seq data validation. Since we intended to explore the expression pattern of the *GjCBP* genes during the pad lamellae development, we selected RNA from pad lamellae of embryos at stages 32, 34, 36, 38, 40, and 42 for transcriptome sequencing, each with three biological replicates. The purified and intact RNA samples were sequenced on the Illumina Novaseq 6000 platform at Sangon Biotech (Shanghai, China). The raw RNA-seq data were deposited in the NCBI Sequence Read Archive (SRA) database under the accession number PRJNA928581 (https://www.ncbi.nlm.nih.gov/bioproject/PRJNA928581).

### Expression level of *GjCBP* genes and differential expression analysis

Subjected to rigorous filtering, raw reads were refined to clean reads for the subsequent analysis: (1) removal of reads with adapters, (2) removal of reads with > 10% N (N denoting unidentified bases), (3) removal of reads with poor quality (those containing over 50% of bases with a base quality Q-value of ≤ 5), and (4) filtering of reads via NGS QC Toolkit v2.3.3 based on a threshold of > 90% reads with Q-value ≥ 20 [69]. The *G. japonicus* reference genome and annotation files (PRJNA899667) were sourced from NCBI. Clean reads were mapped to the reference genome using HISAT2 v2.1.0 (https://daehwankimlab.github.io/hisat2/) [70], with the resulting SAM files being transformed into BAM files and sorted by chromosome positions with Samtools v1.9 (https://github.com/samtools/samtools) [71]. Transcriptomes were assembled and a GTF file with expression information was generated using StringTie v1.3.64 (https://ccb.jhu.edu/software/stringtie/) [72]. CBP gene expression profiles were quantified in terms of TPM values. Aiming to contrast expression patterns of CBP encoding genes with diverse amino acid compositions during the development of epidermal derivatives, cysteine-rich CBPs encoding genes (*ge-cprp-17* to *ge-cprp-26*) and glycine-rich CBP encoding genes (*ge-gprp-17* to *ge-gprp-22*) were chosen haphazardly(Fig. 1). TBtools software (https://github.com/CJ-Chen/TBtools-II/releases) was employed to craft a heatmap, illustrating the varied expression profiles [73]. For differential gene expression analysis, the DESeq2 package (v4.0.5) ( https://bioconductor.org/packages/release/bioc/html/DESeq2.html) in R was used to identify the differentially expressed genes (DEGs) based on the default parameters [74]. To assess the significance of DEGs, a threshold was set where there should be at least a twofold change (|log2 Fold Change| ≥ 1) between two samples, and the *p*-values should be < 0.05 after being adjusted for false discovery rate (FDR). DEGs met these criteria were deemed significant.

### Quantitative real-time PCR (qRT-PCR)

To further explore the molecular mechanisms of GjCBP encoding genes with varied amino acid compositions in setae development, and their expression patterns in different epidermal derivatives. The cDNAs of dorsal epidermis and pad lamellae from stages 32, 34, 36, 38, 40, and 42 embryos and adults were removed from -20 °C, and used to detect the expression patterns of *ge-cprp-17* to *ge-cprp-26* and *ge-gprp-17* to *ge-gprp-22* genes in the developing dorsal epidermis and pad lamellae of embryos via qRT-PCR. Primers for qRT-PCR were designed utilizing Prime Primer 5 software (Table S2), with the *EF-1α* gene of *G. japonicus* (GenBank Accession No. AF199487) serving as the reference gene. The qRT-PCR detection was conducted on a QuantStudio 6 Flex System (Applied Biosystems, Foster City, CA, United States) with SYBR Green Master Mix (Vazyme, Nanjing, China) as the fluorescence dye, following the manufacturer’s instructions. The 2^−ΔΔCT^ method was employed to calculate fold changes of target genes across comparison groups for gene expression analysis [75]. Each experiment was conducted with three biological and three technical replicates, one-way analysis of variance (ANOVA, *p* < 0.05) was applied for statistical analysis. All data are presented as means ± standard error (SE), with error bars symbolizing standard errors across the three biological replicates. Statistical analysis was facilitated by SPSS software (SPSS Inc., Chicago, IL, USA).

### *In situ* hybridization

The study further sought to determine whether the expression patterns of glycine-rich CBPs encoding genes and cysteine-rich CBPs encoding genes differ during the development of various epidermal derivatives [76]. For this, the glycine-rich CBP encoding gene *ge-gprp-19* and the cysteine-rich CBP encoding gene *ge-cprp-17* were chosen haphazardly. Fluorescent in situ hybridization experiments were conducted on the right rear toe lamellae and a small piece of dorsal epidermal tissue of *G. japonicus* embryos at stages 40 and 42, one embryo from each stage. After cleansing, the tissue was immediately submerged in 10% formaldehyde and fixed for 2 to 12 h. Following fixation, a gradient alcohol dehydration process was employed, succeeded by wax immersion for embedding. Paraffin-embedded tissues subsequently sectioned using a microtome. Sections were stretched and dry at 62 °C for 2 h and dewaxed to water. Based on fixation time, the sections were heated in a retrieval solution (Cacl_2_/Tris) for 10 min and naturally cooled. Protease K (20 µg/ml) was applied at 37 °C for 20 min for digestion, tailored to tissue characteristics. This enzymatic step, essential for RNase degradation, was followed by three PBS buffer washes after a pure water rinse. Sections of 4 μm thickness were subsequently stained with a 6 ng/µl probe [77–79]. Add pre-hybridisation solution dropwise and incubate at 37 °C for 1 h. After pouring off the pre-hybridisation solution, probe hybridisation solution containing *ge-gprp-19 + ge-cprp-17* was added dropwise, hybridise overnight at 40 °C in a thermostat. Remove the unbound residual probe hybridisation solution with preheated SSC, add the blocking serum BSA dropwise for 30 min at room temperature, pour off the blocking solution, add mouse anti-DIG-AP dropwise, incubate at 37 °C for 40 min, and then wash with TBS for 4 times. Add BCIP/NBT colour development solution dropwise and rinse with pure water after observation. Cell nuclei were restained using DAPI, and sections were incubated dropwise with DAPI staining solution, protected from light for 8 min, rinsed, and sealed dropwise with an anti-fluorescence quenching sealer. Sections were observed and images were captured under a Nikon orthogonal fluorescence microscope (NIKON ECLIPSE CI), (UV excitation wavelength 330–380 nm, emission wavelength 420 nm, blue light; FAM (488) green excitation wavelength 465–495 nm, emission wavelength 515–555 nm, green light; CY3 red excitation wavelength 510–560 nm, emission wavelength 590 nm, red light.), clear green and red fluorescence signals represent the expression level of the target gene in the tissue. In addition, we highlighted the development of the right rear toe lamellae and setae, as well as cellular differentiation, in order to get to know the developmental status of epidermal derivatives.

### Supplementary Information

Supplementary Material 1: Table S1. All *G. japonicus* samples used for experiments in the article.

Supplementary Material 2: Fig. S1. Amino acid composition of *ge-cprp-17~ge-cprp-26 and ge-gprp-17~ge-gprp-22* genes.

Supplementary Material 3: Table S2. Sequences of primers used in qRT-PCR detection. Table S3. Results of quality control.

Supplementary Material 4: Table S4. Results of mapping.

Supplementary Material 5: Table S5. The number of unigenes.

Supplementary Material 6: Fig. S2. Principal component analysis (PCA) of pad lamellae at different developmental periods. Samples are shown with different labels, closer samples indicate closer expression trends of sample genes.

Supplementary Material 7: Fig. S3. Identification of the differentially expressed genes between different development stages. The horizontal axis is log2(Fold Change) and the vertical axis is -log10(p-value). One dot in the volcano represents one gene, black dots indicate the differentially expressed genes (|log2(Fold Change)| ≥ 1, p-value ≤ 0.05). A (34 vs 32), B (36 vs 32), C (38 vs 32), D (40 vs 32), E (42 vs 32), F (36 vs 34), G (38 vs 36), H (40 vs 38), I (42 vs 40).

Supplementary Material 8: Table S6. The mean TPM value of *GjCBP *genes in RNA-seq detection.

Supplementary Material 9.

## Data Availability

The datasets supporting the conclusions of this article are available in the [NCBI Sequence Read Archive under project PRJNA928581] repository (https://www.ncbi.nlm.nih.gov/bioproject/PRJNA928581), this article contains supplementary figures and tables, which are available to authorized users.
