# Supplementary figures and images for: Spatio-temporal expression patterns of glycine-rich beta proteins and cysteine-rich beta proteins in setae development of Gekko japonicus
Source: BMC Genomics. 2024 May 31;25:535. doi: 10.1186/s12864-024-10426-8 (PMC11140998; doi:10.1186/s12864-024-10426-8)

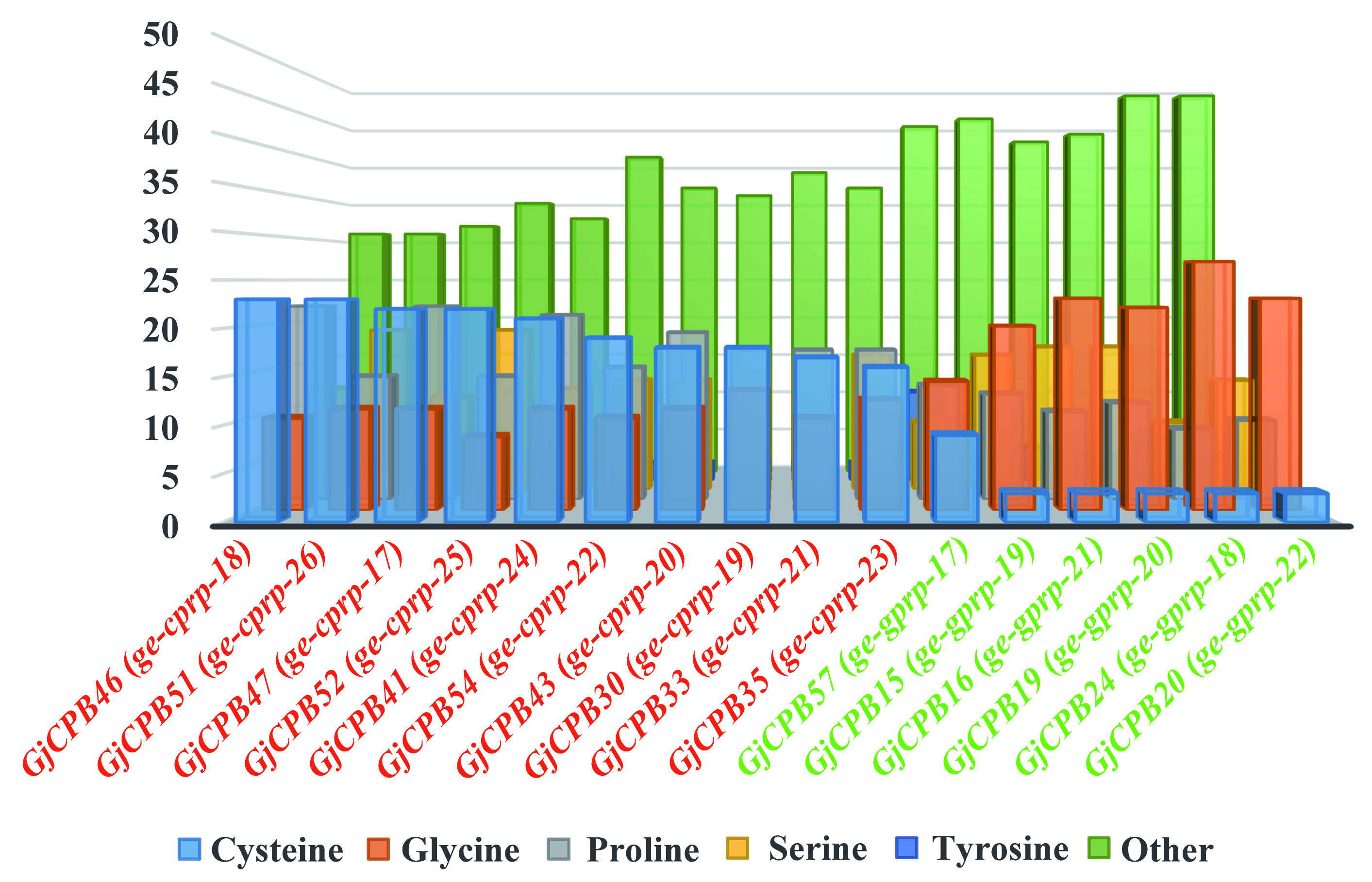

Supplement: Supplementary file 1 — Supplementary Material 1: Table S1. All G. japonicus samples used for experiments in the article. [file 12864_2024_10426_MOESM1_ESM.tif]

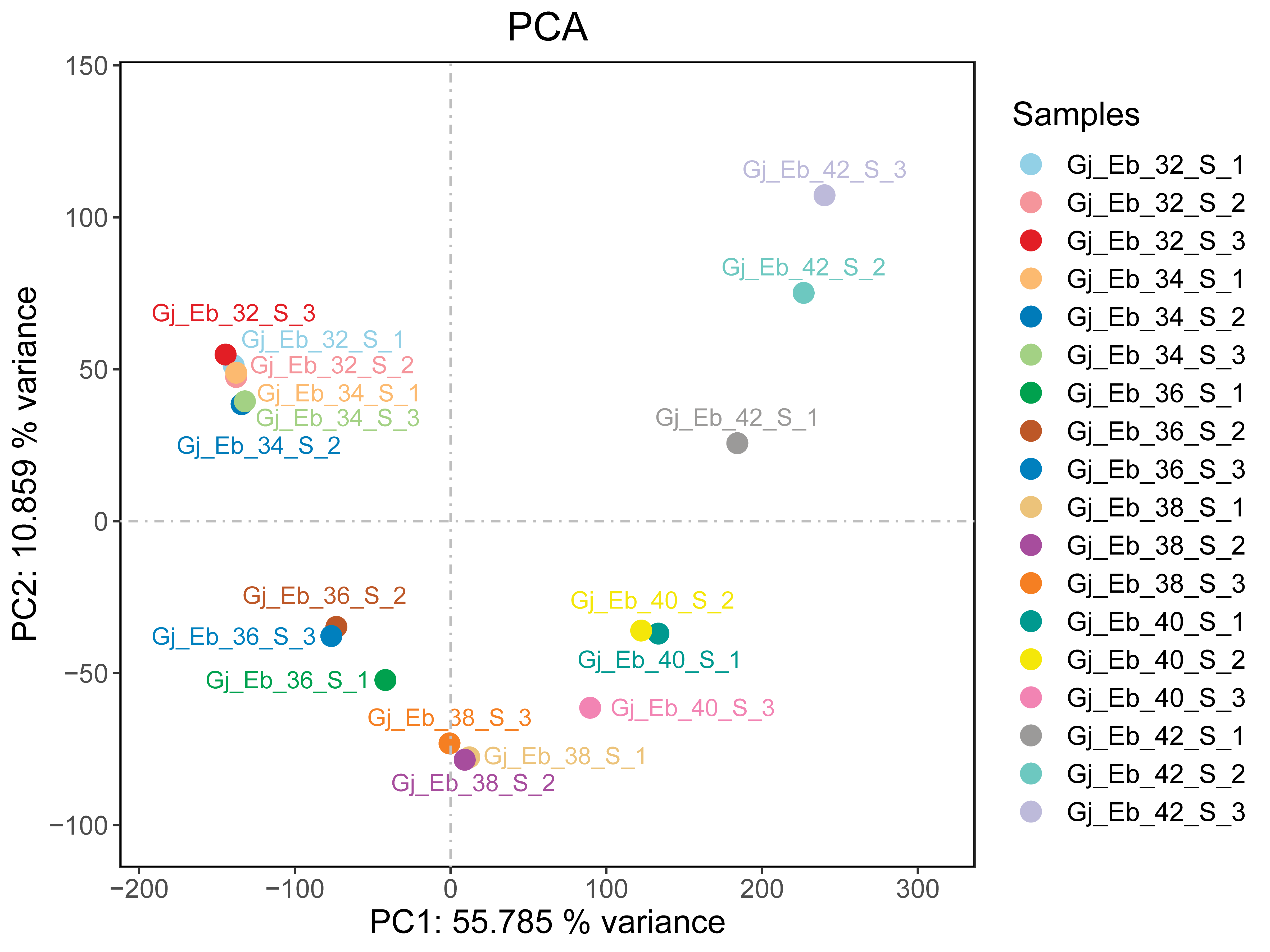

Supplement: Supplementary file 2 — Supplementary Material 2: Fig. S1. Amino acid composition of ge-cprp-17~ge-cprp-26 and ge-gprp-17~ge-gprp-22 genes. [file 12864_2024_10426_MOESM2_ESM.tif]
