# Supplementary material for: Spatio-temporal expression patterns of glycine-rich beta proteins and cysteine-rich beta proteins in setae development of Gekko japonicus
Source: BMC Genomics. 2024 May 31;25:535. doi: 10.1186/s12864-024-10426-8 (PMC11140998; doi:10.1186/s12864-024-10426-8)
Supplement: Supplementary file 4 — Supplementary Material 4: Table S4. Results of mapping. [file 12864_2024_10426_MOESM4_ESM.docx]

Table S1. All *G. japonicus* samples used for experiments in the article

| samples | Number of embryos | Purpose |
| --- | --- | --- |
| Adult | 60 | Embryos collection |
| Adult | 3 | RNA extraction |
| Embryos | 18 | RNA extraction |
| Embryos | 18 | Morphological observation |
| Embryos | 2 | *In situ* hybridization |
