# Supplementary material for: Spatio-temporal expression patterns of glycine-rich beta proteins and cysteine-rich beta proteins in setae development of Gekko japonicus
Source: BMC Genomics. 2024 May 31;25:535. doi: 10.1186/s12864-024-10426-8 (PMC11140998; doi:10.1186/s12864-024-10426-8)
Supplement: Supplementary file 5 — Supplementary Material 5: Table S5. The number of unigenes. [file 12864_2024_10426_MOESM5_ESM.docx]

Table S2. Sequences of primers used in quantitative real-time (qRT-PCR) detection.

| Primers | Sequence (5’→3’) |
| --- | --- |
| *EF-1α*-F | AAGACCCACATCAACATCG |
| *EF-1α*-R | CAGCCGCTTCCTTCTCAAA |
| *ge-gprp-17*-F | ACCCATCCTGAACTGCTACAA |
| *ge-gprp-17*-R | CACCACAACCATATCCTCCTC |
| *ge-gprp-18*-F | ATAAAAAGGCTCCCTGGGCTC |
| *ge-gprp-18*-R | ACAGCAGGGCCATACCCATA |
| *ge-gprp-19*-F | TCCTCAGAGAAGCAGACACA |
| *ge-gprp-19*-R | CCCCAGAGTTGTGGCTGAAA |
| *ge-gprp-20*-F | GGCAGTGGTCTTTATGGTGGA |
| *ge-gprp-20*-R | ATAGGACCGCTTCCCTCTGT |
| *ge-gprp-21*-F | GGTCCCGGCAGAGTGATTTC |
| *ge-gprp-21*-R | ATAGGACCGCTTCCCTCTGT |
| *ge-gprp-22*-F | TCATCTCTCTCATTCTCTTGCCT |
| *ge-gprp-22*-R | CGCATGATGGAATGGCGAAG |
| *ge-cprp-17*-F | TTGTACCGTTGTGGTCCCAG |
| *ge-cprp-17*-R | TCGTGTGAAGCTCCCCTTTC |
| *ge-cprp-18*-F | TGCTGCGGTAACTCAACATCAG |
| *ge-cprp-18*-R | CTGGGACCACAACGGTACA |
| *ge-cprp-19*-F | CCATGTGGATACCCTGTCGG |
| *ge-cprp-19*-R | TGGGATCACGACGGTTGAAG |
| *ge-cprp-20*-F | TATAGGACGGATCGACGGCT |
| *ge-cprp-20*-R | CCCACAGAGGGAAGACGTTC |
| *ge-cprp-21*-F | TGTGACTACCCAAAAGGCGG |
| *ge-cprp-21*-R | TGGCCCTGGGACTGTAACAA |
| *ge-cprp-22*-F | ACAACTTCGTGGACTTGGGG |
| *ge-cprp-22*-R | ATCAGACGTTGGTGGTCACG |
| *ge-cprp-23*-F | CATGTGACTACCCAAAAGGCG |
| *ge-cprp-23-*R | GGGATCACGACGGTTGAAGG |
| *ge-cprp-24*-F | TGCATCAACCAGATCCCACC |
| *ge-cprp-24*-R | CCGACACTCAAGTCTGTGGA |
| *ge-cprp-25*-F | GGCCCACAACGTTCTTCCTA |
| *ge-cprp-25*-R | AGTGGTTTGTCCACTTCCCG |
| *ge-cprp-26*-F | ACTTCTCCGCGAAACAGACTC |
| *ge-cprp-26*-R | CGGATCCGATCAATGTGCCA |
